# Beyond Blood Pressure: Emerging Pathways and Precision Approaches in Hypertension-Induced Kidney Damage

**DOI:** 10.3390/ijms26157606

**Published:** 2025-08-06

**Authors:** Charlotte Delrue, Marijn M. Speeckaert

**Affiliations:** 1 Department of Nephrology, Ghent University Hospital, 9000 Ghent, Belgium; charlotte.delrue@ugent.be; 2 Research Foundation-Flanders (FWO), 1000 Brussels, Belgium

**Keywords:** hypertensive kidney disease, pathways, renin–angiotensin–aldosterone system

## Abstract

Recent studies have demonstrated that the development and progression of hypertensive kidney injury comprise not only elevated systemic blood pressure but also a complex interplay of cellular, molecular, and genetic mechanisms. In this report, we outline the key emerging pathways—ranging from dysregulated renin–angiotensin system signaling, oxidative stress, immune-mediated inflammation, and metabolic abnormalities to epigenetic alterations and genetic susceptibilities—that contribute to kidney damage in hypertensive conditions. In addition, we also discuss precision medicine approaches like biomarker-directed therapies, pharmacologically targeted therapies, and device-based innovations for modulating these pathways. This integrative review emphasizes the application of omics technologies and genetically guided interventions to better stratify patients and offer personalized care for hypertensive kidney disease.

## 1. Introduction

Hypertension remains one of the leading global risk factors for end-stage kidney disease (ESKD) and chronic kidney disease (CKD) and is a cause of preventable morbidity and mortality worldwide. Approximately 1.28 billion adults worldwide have hypertension, with approximately 10–15% of CKD cases being directly caused by hypertensive vasculature damage [1,2]. While the conventional paradigm is that of elevated systemic blood pressure leading to glomerular hyperfiltration and arteriolosclerosis, recent evidence has elucidated a much more intricate pathophysiological milieu with a wide range of hemodynamic, molecular, immunological, metabolic, and genetic factors. In addition to mechanical stress, hypertensive kidney injury is a progressive, multifactorial disease characterized by repeated activation of the renin–angiotensin–aldosterone system (RAAS), chronic inflammation, oxidative stress, and immune dysregulation. Cellular stress reactions in renal tubular epithelial cells, glomerular podocytes, and vascular endothelial cells are enhanced by mitochondrial bioenergetic perturbations, epigenetic marks (e.g., microRNAs and DNA methylation), and incorrect crosstalk between renal and immune cells [3,4,5]. These pathological processes result in tubular atrophy, interstitial fibrosis, and glomerulosclerosis, commonly preceding obvious declines in the glomerular filtration rate (GFR).

Most concerning is the evidence that the majority of these processes begin far in advance of hypertension being clinically apparent, reinforcing the concept of “prehypertensive kidney injury” [6]. For example, early oxidative stress can inhibit nephroprotective nitric oxide (NO) pathways, disrupt endothelial function, and prime fibrogenesis. Similarly, immune responses, such as the expansion of Th17 cells and activation of the NOD-, LRR-, and pyrin domain-containing protein 3 (NLRP3) inflammasome, have been shown to exacerbate hypertension-induced nephropathy in animal models and are being explored in human populations [7,8,9]. Most notably, not all hypertensive individuals sustain progressive kidney damage; hence, the value of individual susceptibility. Genome-wide association studies and transcriptomic profiling have implicated several loci, including *ADD3*, *DUSP5*, and *UMOD*, which influence renal hemodynamics, tubular reabsorption of sodium, and inflammatory setpoints [10,11,12]. Furthermore, epigenetic suppression of antioxidant protective genes (e.g., *Nrf2*) and activation of fibrogenic pathways by transforming growth factor-beta (TGF-β) have emerged as significant inducers of inter-individual heterogeneity in disease progression [13]. In response to these findings, the field is now heading toward precision nephrology, an approach that integrates omics-based molecular profiling, biomarker-based risk stratification, and individualized therapeutic regimens. High-throughput technologies, such as single-cell RNA sequencing, urinary proteomics, and plasma metabolomics, now allow for the characterization of discrete molecular subtypes of hypertensive kidney disease. These subtypes can exhibit differential sensitivity to therapeutic agents, such as sodium–glucose cotransporter 2 (SGLT2) inhibitors, mineralocorticoid receptor antagonists, and new immunomodulators [14,15,16].

This review aims to provide a comprehensive overview of the novel molecular pathways underlying hypertension-induced renal damage and to discuss precision medicine strategies beyond conventional blood pressure reduction. By closely examining the intersection of immune signaling, metabolic reprogramming, and vascular remodeling in the hypertensive kidney, we aspire to map a plan for future research and translation to the bedside. Finally, the translation of these discoveries into practice can revolutionize the management of hypertensive kidney disease, allowing for earlier detection and personalized treatment with improved outcomes.

## 2. Emerging Molecular Pathways Beyond Hemodynamics

The pathogenesis of hypertensive kidney disease is a multifaceted intersection of molecular mechanisms. Table 1 summarizes the key biological processes, including hemodynamic stress, immune activation, oxidative injury, and metabolic dysregulation, as well as the key mediators that have been defined in experimental and clinical models (Table 1).

### 2.1. Renin–Angiotensin–Aldosterone System

**Figure 1:**
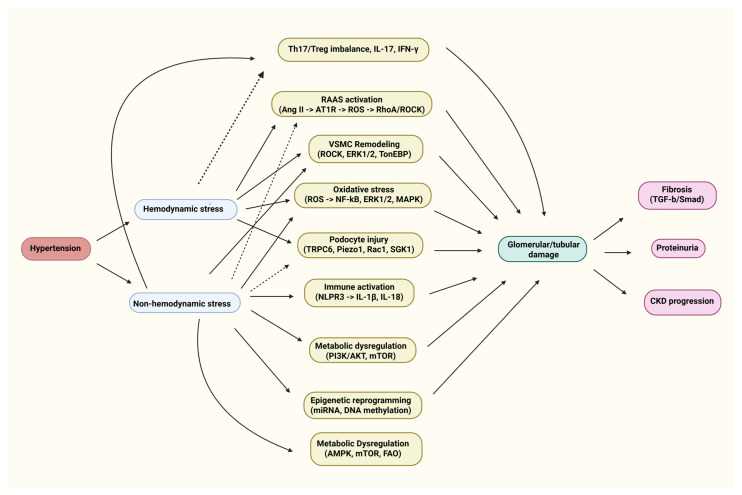
Integrated molecular and cellular mechanisms linking hypertension to kidney injury and chronic kidney disease progression: Hypertension leads to both hemodynamic and a series of non-hemodynamic stressors, which induce damage to the glomeruli and tubules of the kidney. Hemodynamic stress induced by increased intraglomerular pressure (involving increased mechanical stress) results in the activation of pathogenic pathways associated with the RAAS and oxidative stress, VSMC remodeling, and podocyte injury mediated via mechanosensitive channels (e.g., TRPC6 and Piezo1). Therefore, these mediators enhance immune activation above baseline levels by shifting the balance of immune responses to promote Th17/Treg imbalance and cytokine secretion associated with Th17/Treg activation (e.g., IL-17 and IFN-γ). Alternatively, the non-hemodynamic stressors represent inflammatory, metabolic, and epigenetic changes that amplify oxidative stress and can promote direct podocyte injury and pro-inflammatory activation of the immune response through the NLRP3 inflammasome and activation of pro-inflammatory cytokines (e.g., IL-1β and IL-18). In this case, metabolic dysregulation was related to dysregulation of mTOR and AMPK signaling, defective fatty acid oxidation, and impaired glycolysis. Epigenetic changes in kidney injury are associated with DNA methylation and microRNA dysregulation (e.g., miR-29b and miR-155), which enhance fibrosis and dysfunction of the cells. The integration of these mechanisms generates glomerular and tubular damage, which aids in downstream pathological ramifications, including renal fibrosis (via TGF-β/Smad signaling), proteinuria, and CKD progression. Solid arrows indicate direct mechanistic relationships, whereas dashed arrows indicate contributory or feedback relationships. Abbreviations: AMPK, AMP-activated protein kinase; CKD, chronic kidney disease; FAO, fatty acid oxidation; IFN-γ, interferon-gamma; IL-1β, interleukin-1 beta; IL-17, interleukin-17; IL-18, interleukin-18; miR-29b, microRNA-29b; miR-155; microRNA-155; NLRP3, NOD-, LRR-, and pyrin domain-containing protein 3 (inflammasome); mTOR, mechanistic target of rapamycin; Piezo1, piezo-type mechanosensitive ion channel component 1; RAAS, renin–angiotensin–aldosterone system; Smad, mothers against decapentaplegic homolog (signaling proteins); TGF-β, transforming growth factor beta; Th17, T helper 17 cell; Treg, regulatory T cell; TRPC6, transient receptor potential cation channel subfamily C member 6; VSMC, vascular smooth muscle cell.

The RAAS is the key pathophysiological pathway for hypertensive kidney damage, both by its actions on hemodynamics and by a vast array of deleterious cellular and molecular effects documented in experimental models. In vivo animal studies demonstrate that chronic angiotensin II (Ang II) infusion leads to glomerular hypertension, podocyte damage, and increased albuminuria through both direct AT1R-dependent pathways and secondary inflammation and oxidative stress [22]. Angiotensin II (Ang II), the principal bioactive RAAS peptide, has its effects mediated by binding to the angiotensin II type 1 receptor (AT1R) on many renal and vascular cells, which leads to vasoconstriction, sodium reabsorption, and release of aldosterone (Figure 1). However, beyond these systemic effects, Ang II triggers substantial kidney injury through increased Ras-related C3 botulinum toxin substrate 1 (Rac1)-mediated activation of mineralocorticoid receptors (MRs), even in the absence of elevated aldosterone levels. In double-transgenic hypertensive mouse models, increased salt intake “exacerbated” Ang II-induced kidney damage mediated by this Rac1-MR axis, which manifested as glomerulosclerosis and proteinuria, a process that was prevented by both Rac1 inhibitors and MR antagonists, but not simply by lowering blood pressure [23].

**Figure 2:**
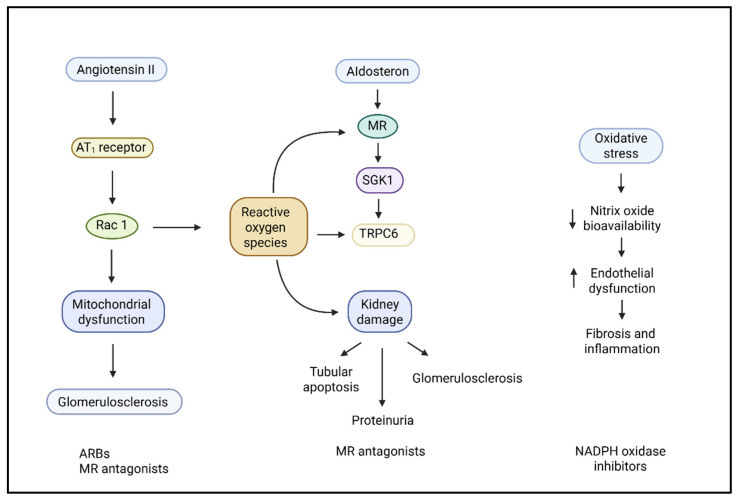
Relationship between the renin–angiotensin–aldosterone system (RAAS) and oxidative stress in hypertensive kidney injury: This illustration provides a schematic of the molecular interplay between RAAS activation and oxidative stress and the resulting hypertensive kidney injury. Angiotensin II (Ang II), the primary effector of the RAAS, engages the angiotensin II type 1 receptor (AT1R) on vascular and/or renal cells and rapidly activates multiple downstream pathways, including those that also activate nicotinamide adenine dinucleotide phosphate (NADPH) oxidases (NOX enzymes), which produce reactive oxygen species (ROS). ROS will increase vasoconstriction, impair endothelial function, and lead to mitochondrial injury. Additionally, ROS trigger cell signaling cascades with redox-sensitive cascades, e.g., nuclear factor kappa-light-chain-enhancer of activated B cells (NF-κB) and mitogen-activated protein kinases (MAPKs), causing inflammation and fibrosis. Aldosterone can similarly increase oxidative stress and tissue injury by activating mineralocorticoid receptors (MRs). These poorly controlled increases in oxidant stress, RAAS activation, and inflammation produce a spiral of kidney injury and fibrosis that results from a self-perpetuating feedback loop.

Experimental and clinical studies have shown that Ang II induces podocyte injury in part by promoting intracellular Ca^2+^ influx and ROS production via NADPH oxidase and TRPC6 channels, processes controlled by the activation of nicotinamide adenine dinucleotide phosphate (NADPH) oxidases and transient receptor potential canonical 6 (TRPC6) channels. These changes lead to actin cytoskeleton destabilization and the effacement, detachment, and apoptosis of podocytes, which are essential contributors to proteinuria and glomerulosclerosis (Figure 2) [24,25]. Fibroblast growth factor receptor 1 (FGFR1) signaling in tubular epithelial cells has also emerged as a novel downstream effector of Ang II, promoting fibrogenesis through signal transducer and activator of transcription 3 (STAT3) activation and extracellular matrix deposition [22].

Moreover, Ang II induces extracellular matrix accumulation and mesangial cell proliferation via the overexpression of TGF-β and connective tissue growth factor (CTGF), thereby initiating mesangial growth and glomerular fibrosis [26]. Ang II also induces the activation of pro-inflammatory transcription factors, such as nuclear factor kappa-light-chain-enhancer of activated B cells (NF-κB), which supports the expression of cytokines and chemokines such as IL-6, monocyte chemoattractant protein-1 (MCP-1), and tumor necrosis factor-alpha (TNF-α) and promotes immune cell infiltration [27].

Aldosterone, via the MR, amplifies kidney injury. While previously associated with sodium retention and volume overload, aldosterone also boosts oxidative tension and inflammation through non-genomic mechanisms. Through MR activation in renal tubular epithelial cells and vascular endothelial cells, the increased production of mitochondrial ROS and greater expression of pro-fibrotic genes stimulate tubulointerstitial fibrosis [28]. The MR not only promotes oxidative stress in the kidneys but also contributes to oxidative stress throughout the body by promoting mitochondrial dysfunction, particularly under pathological conditions such as obesity, diabetes, and hyperaldosteronism. MR activation disrupts mitochondrial quality control by inhibiting biogenesis, enhancing fission, and inhibiting mitophagy, resulting in damaged mitochondria and excessive ROS production. The MR also initiates the p53-p21 senescence pathway and decreases sirtuin levels [29]. Non-genomic MR signaling activates both NADPH oxidase and Rac1 GTPase, resulting in excessive ROS production and enhanced transcriptional activity of MRs [30]. MR activation by aldosterone activates MAPK/p38 signaling, which decreases mitochondrial DNA content and ATP synthesis and increases oxidative stress, which is reversible with MR antagonists or antioxidants [31]. Moreover, aldosterone induces endothelial dysfunction by reducing the bioavailability of nitric oxide (NO) and increasing the expression of endothelin-1, thereby enhancing vascular stiffness and impairing renal perfusion [32]. These actions place aldosterone and MR signaling in the middle ground of hemodynamic and non-hemodynamic damage in hypertensive nephropathy. This dysregulation of hormones does not operate independently in a clinical entity. In patients with CKD, a complicated self-amplifying circular process occurs with (1) decreased nephron number, (2) sodium retention, (3) stimulation of the RAAS, and (4) increased sympathetic nervous system activity. All of these variables have a multifaceted interplay. As renal mass decreases, sodium excretion is inhibited, and volume expansion then activates both RAAS and sympathetic pathways. In CKD subjects, increased renal sympathetic nerve activity is associated with increased tubular sodium reabsorption and interacts directly with resistant hypertension [33,34]. Sympathetic stimulation also stimulates the sodium–chloride cotransporter (NCC), which is involved in sodium retention and perhaps in age-related increased blood pressure [35]. Recent insights into precision hypertension further underscore that these pathophysiological responses vary between individuals, based on genetic background, hormonal profiles, and sodium sensitivity. Such variability highlights the need for individualized approaches in managing hypertensive CKD, as these overlapping mechanisms do not contribute equally in all patients [36]. These processes combined contribute to an accelerated worsening of hypertension and kidney function in CKD patients.

Therapeutically, RAAS blockade using angiotensin-converting enzyme inhibitors (ACEIs) or angiotensin receptor blockers (ARBs) remains the cornerstone of treatment for hypertension-related kidney disease. They have been shown to reduce proteinuria, slow the progression of CKD, and reduce glomerular pressure [37]. Notably, multiple clinical studies have confirmed that these agents confer renoprotection beyond their effects on blood pressure. For instance, both ACEIs and ARBs have been associated with reductions in proteinuria, slower GFR decline, and delayed progression to kidney failure in hypertensive and diabetic populations [38,39], and their protective effects have been observed even when systolic blood pressure targets were similarly achieved across treatment groups [40].

Interestingly, recent results indicate that ACE inhibitors, such as captopril, might mitigate inflammation not only via hemodynamic effects but also by modulating anti-aging pathways. Captopril has been shown to inhibit angiotensin-converting enzyme-related carboxypeptidase-1 (ACN-1), an enzyme homologous to the angiotensin-converting enzyme, and modulate dauer and longevity by inhibiting ACN-1 in *Caenorhabditis elegans*. This adds to the idea that inflammation could be a product of age-related molecular dysfunction rather than a cause of it [41]. More recently, the introduction of non-steroidal MR antagonists, such as finerenone, has provided a promising therapeutic alternative with a superior safety profile compared to older drugs, such as spironolactone. Finerenone has been found to be effective in reducing kidney fibrosis and inflammation, particularly in patients with diabetes and hypertension who are co-affected, and it has been found to reduce albuminuria and prevent the worsening of kidney function [42,43].

Apart from the RAAS’s effects on renal sodium reabsorption, the epithelial sodium channel (ENaC) is an often ignored but central component to the pathogenesis of hypertension and the renal sequelae from hypertension. ENaC, predominantly expressed in the apical membrane of distal nephron principal cells, is a crucial component in the regulation of sodium reabsorption as well as the fine-tuning of extracellular volume status. Under normal conditions, ENaC is tightly regulated by aldosterone and the MR, which enhance the transcription of and α-, β-, and γ-subunits into the apical membrane of the ENaC channel to enhance sodium reabsorption and increase potassium excretion [44]. Under pathological conditions, ENaC is a key effector of aldosterone-mediated hypertensive injury, particularly under resting and MR overactivation, as well as dysregulated, aldosterone-independent pathways. Notably, angiotensin II has been shown to directly induce ENaC expression through MR-independent signaling, involving Rac1 activation and serum and glucocorticoid-regulated kinase 1 (SGK1) activation, which both induce stabilization of ENaC on the apical membrane by preventing ubiquitin-mediated degradation via Nedd4-2 [45,46]. As a result, there is continued sodium retention, volume expansion, and increased blood pressure—and, thus, renal hemodynamic stress. Furthermore, pro-inflammatory cytokines like interleukin (IL)-17A and TNF-α have been shown to upregulate ENaC expression and function in tubular epithelial cells, linking immunological activation to increased sodium reabsorption and salt-sensitive hypertension [47,48]. The immune–tubular axis represents a nexus of inflammatory and epithelial transport abnormalities associated with the hypertensive kidney. Also, ENaC activation is synergistically modulated by oxidative stress, whereby reactive oxygen species (ROS) can synergistically promote the channel open probability, thus further linking it into the oxidative–inflammatory paradigm of hypertensive nephropathy [49]. Moreover, in addition to abnormal ENaC expression and activity in the distal nephron, it has also been shown to be expressed in non-classical sites such as in endothelial and immune cells, where it contributes to vascular stiffness, endothelial dysfunction, inflammation, and even regulation of inflammasomes. Immune cells, especially macrophages, upregulate ENaC and have ENaC-mediated sodium influx as a requirement for NOD-, LRR-, and pyrin domain-containing protein 3 (NLRP3) inflammasome activation, and eventually, secretion of IL-1β, which can lead to or worsen renal inflammation [50]. These data broaden the conceptual function of ENaC from solely an epithelial sodium transporter to a multifaceted effector of hypertensive injury across organ systems. In terms of therapy, ENaC inhibition (amiloride) has demonstrated pressor-lowering capacity and renoprotective ability, particularly in salt-sensitive models or in those of Liddle syndrome [49]. In experimental models of hypertension, amiloride significantly reduces borrowing but also reduces markers of oxidative and inflammatory injury in the kidney, with both hemodynamic and molecular effects. This has prompted renewed interest in the use of ENaC blockers in the treatment of volume-dependent and inflammatory hypertensive kidney disease [51].

### 2.2. Oxidative Stress and Reactive Oxygen Species

Chronic hypertension is a major contributor to oxidative stress, which is a major mechanism of the deterioration of hypertensive kidney disease. The primary source of ROS in hypertensive kidney disease is Ang II, which activates NADPH oxidase isoforms (NOX1, NOX2, and NOX4) via the AT1 receptor. Ang II stimulation causes excessive generation of superoxide anions and hydrogen peroxide, which contribute to vasoconstriction, endothelial dysfunction, and vascular remodeling [52]. The oxidative burden increases glomerular hypertension and mesangial cell growth and causes podocyte damage, which leads to glomerulosclerosis and tubulointerstitial fibrosis. Furthermore, ROS will react rapidly with nitric oxide (NO) to form peroxynitrite, a highly reactive oxidant that diminishes NO bioavailability, impairs vasodilation, and promotes a pro-inflammatory and pro-thrombotic vascular environment [53]. In the kidney, redox imbalance can severely disrupt autoregulation and tubuloglomerular feedback, the two mechanisms that help to maintain constant glomerular perfusion. ROS disturb these mechanisms, mainly in the afferent arterioles, subsequently resulting in higher intraglomerular pressures and mechanical stresses. ROS may also upregulate adhesion molecules like intercellular adhesion molecule 1 (ICAM-1) and vascular cell adhesion molecule 1 (VCAM-1) on endothelial cells, allowing leukocyte recruitment and the start of sterile inflammation [53,54,55,56].

**Figure 3:**
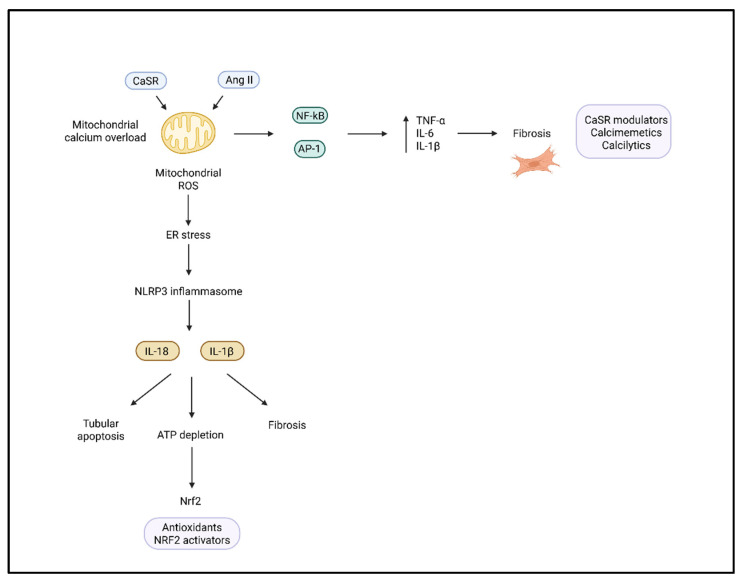
Mitochondrial dysfunction in hypertensive kidney disease: This figure summarizes the role of mitochondrial dysfunction in hypertensive nephropathy. In the hypertensive state, calcium-sensing receptor (CaSR) and angiotensin II (Ang II) stimulate the mitochondrial calcium overload, which leads to the production of mitochondrial reactive oxygen species (mtROS). The mtROS result in the release of nuclear factor kappa-light-chain-enhancer of activated B cells (NF-κB), activator protein-1 (AP-1), etc., which can induce pro-inflammatory cytokines such as tumor necrosis factor-alpha (TNF-α), interleukin (IL)-6, and IL-1β. Mitochondrial injury promotes endoplasmic reticulum (ER) stress and triggers an intrinsic pro-inflammatory program that includes activation of the NOD-, LRR-, and pyrin domain-containing protein 3 (NLRP3) inflammasome. This results in caspase-1 activation and the release of IL-1β and IL-18. The final common pathway is tubular apoptosis, ATP depletion, and fibrosis, specifically in the ischemia-sensitive part of the nephron. The antioxidant program that is typically implemented by the host, including the nuclear factor erythroid 2-related factor 2 (Nrf2) pathway, is impaired, leading to redox dysregulation. Targetable therapeutic agents include Nrf2 activators, mitochondrial antioxidants [mitoquinone (MitoQ)], and modulators of the CaSR (either calcimimetics or calcilytics) that may lead to mitigation of this pathological process and renal preservation.

Mitochondrial dysfunction represents both a source and a target of oxidative damage. In the hypertensive kidney, mitochondrial dysfunction can result in excess production of mitochondrial reactive oxygen species (mtROS), which activate redox-sensitive transcription factors like NF-κB, activator protein 1 (AP-1), and others that drive the expression of pro-inflammatory cytokines (e.g., TNF-α, IL-6, and IL-1β), contributing to a microenvironment that favors renal injury. In areas of the kidney that are prone to ischemia, like the renal medulla, this is coupled with additional tubular apoptosis and ATP depletion, fibrosis, etc. (Figure 3) [57,58].

Oxidative stress also inhibits central antioxidant defenses. For example, the endogenous antioxidant defense system, consisting of superoxide dismutases (SOD1 and SOD2), glutathione peroxidase (GPx), and catalase, is frequently downregulated in hypertensive states. Furthermore, the nuclear factor erythroid 2-related factor 2 (Nrf2) is often inhibited, and Nrf2 activates antioxidant gene expression via antioxidant response elements (AREs), continuing to shift the balance towards oxidative stress and cellular damage [59].

Experimental animal models provide strong evidence supporting the central role of oxidative stress in hypertensive kidney injury. Spontaneously hypertensive rats (SHRs) exhibit elevated renal expression of NADPH oxidases and biomarkers, such as NGAL, which correlates with histological findings of glomerulosclerosis and tubulointerstitial fibrosis [60]. Hypertensive rodent models have been studied for chronic radiotelemetry, which has demonstrated that blood pressure control is the best overall predictor of kidney protection, and the importance of the class of pharmacological agents being examined is decreasing, particularly when considering a load-dependent oxidative injury model [61]. More recently, progressive CKD with hypertension, inflammation, and oxidative injury has been observed in the C57BL/6 5/6 nephrectomy model. With this model, significant fold increases in pro-inflammatory cytokines (such as TNF-α and IL-1β), as well as a change in redox and mitochondrial dysfunction, were demonstrated, making the C57BL/6 nephrectomy model more similar to the progression of the disease as seen in humans [62]. Importantly, this model displays oxidative stress, and antioxidant treatments, such as Nrf2 activators or tempol, display renoprotective effects in all CKD models and support the notion that oxidative stress is a pathological driver and potentially a therapeutic target. Two recent human studies have provided strong evidence that, in hypertensive kidney disease, the processes of oxidative stress and sterile inflammation are mechanistically linked. Gao et al. presented biopsy-based evidence showing that mitochondrial DNA (mtDNA) is released following hypertensive injury, and that this mtDNA activates the stimulator of interferon genes (STING) signaling pathway in renal tubular epithelial cells. This mtDNA-STING signaling connects the acute innate immune response [e.g., releasing type I interferon, activating nuclear factor kappa-light-chain-enhancer of activated B cells (NF-κB) transcription factor, and expressing fibrotic genes] and accelerates the response to kidney injury. These findings underscore the role of mitochondrial injury as an upstream stimulator of inflammation through DNA-sensing pathways in humans [63]. In addition, non-hemodynamic mechanisms, such as mitochondrial dysfunction, endoplasmic reticulum (ER) stress, and redox imbalance, are involved in the hypertension-induced renal pathology. Oxidative stress in human kidneys is not just a secondary effect of hypertension but a central mediator of renal inflammation and fibrosis [64].

The calcium-sensing receptor (CaSR) is a type C GPR, broadly expressed in renal tubular epithelial cells, podocytes, vascular smooth muscle cells, and innate immune cells. The CaSR is an essential regulator of inflammation and tissue injury in hypertensive kidney disease. The CaSR is activated by either extracellular calcium or the binding of calciprotein particles. CaSR activation will cause intracellular calcium release through the phospholipase C-inositol 1,4,5-trisphosphate (PLC-IP_3_) signaling pathway, which drives calcium overload in mitochondria and leads to endoplasmic reticulum stress and ROS generation [65,66]. These signaling events cooperate, leading to NLRP3 activation in macrophages and monocytes, which partially stimulates caspase-1. Downstream of all of this is the release of the pro-inflammatory cytokines IL-1β and IL-18 [67,68], and ultimately tubulointerstitial inflammation and fibrosis. In SHR models, CaSR downregulation is associated with endothelial dysfunction, increased vasoconstriction, and activation of the RAAS, creating a feedback loop that results in vascular stiffness, hypertension, and renal hypoperfusion [69]. Additionally, the CaSR also regulates sodium and calcium reabsorption in the thick ascending limb and the collecting duct. Therefore, it is a key integrator of hemodynamic and electrolyte-driven stress [70]. Pharmacological modulation of CaSR offers a novel therapeutic opportunity. In preclinical models, calcimimetics such as R-568 or NPS R-467 enhance CaSR activity, attenuate vascular injury, and reduce oxidative stress, while calcilytics like Calhex231 inhibit CaSR-mediated mitochondrial calcium overload and NLRP3 activation (Figure 3) [65,71]. These interventions not only reduce proteinuria and preserve the GFR but also limit immune infiltration and fibrosis, supporting a pathophysiological role for the CaSR at the intersection of oxidative, immune, and tubular injury in hypertension-induced nephropathy.

Given these mechanisms, antioxidant therapies have been tested to see if they can reduce oxidative stress in hypertensive kidney disease. Bardoxolone methyl is one of the most studied agents to date. It is a synthetic triterpenoid that activates the gene transcription of antioxidant response genes by promoting the transcription of Nrf2. Earlier studies (i.e., BEAM and TSUBAKI) showed improvements in GFR in patients with diabetic CKD. However, bardoxolone methyl is still under investigation in hypertensive nephropathy and CKD [72]. N-acetylcysteine (NAC) may be another potential agent, as it is a precursor of glutathione and a free radical scavenger. NAC has primarily been studied in contrast-induced nephropathy; however, small studies indicate a possible role in reducing proteinuria and markers of oxidative stress in patients with hypertensive CKD [73]. Allopurinol, a xanthine oxidase inhibitor that can decrease uric acid-induced ROS production, can lead to the reduction in blood pressure in hypertensive adolescents and adults with early stages of renal impairment, and it may improve endothelial function in these populations as well [74]. Apart from classical antioxidants, potential agents such as tempol (a superoxide dismutase mimetic) and mitochondria-targeted antioxidants such as MitoQ have been documented to result in renoprotective effects in animal models of hypertension, but they remain largely experimental in humans [75]. Current human clinical approaches are focused on indirect antioxidant mechanisms through the blockade of RAAS, SGLT2 inhibitors, and MR antagonists, which also have the potential to suppress oxidative signaling pathways. While the signals appear encouraging, clinical trials have yet to demonstrate that antioxidant monotherapy should become a standard of care in hypertensive kidney disease, emphasizing the need for better biomarker-directed strategies as well as long-term outcome measures.

### 2.3. Immune and Inflammatory Mechanisms

There is now strong and mounting evidence that immune responses, and particularly those mediated by adaptive immunity, play a role in mediating kidney injury in hypertension. Experimental models have shown that hypertensive stimuli, such as Ang II or salt loading, cause T-cell infiltration into the kidneys, with oligoclonal expansion and activation of T lymphocytes. In both murine and rat models, CD3+ T cells accumulate in the renal tissue within days of exposure to hypertensive stimuli, accompanied by the upregulation of adhesion molecules such as ICAM-1 and VCAM-1 on the renal vasculature, promoting immune cell entry [76].

This comprises key co-stimulatory pathways, such as CD80/CD86–CD28, which play a crucial role in T-cell activation and proliferation. Dendritic cells exposed to Ang II or oxidative stress enhance the expression of CD80 and CD86 and present neoantigens, such as isolevuglandin-modified proteins, amplifying autoreactive T-cell responses and perpetuating local inflammation [77]. These T cells also secrete pro-inflammatory cytokines such as IL-6, TNF-α, and interferon-gamma (IFN-γ), which exacerbate renal inflammation and injury [78,79]. These cytokines also reduce endothelial nitric oxide synthase (eNOS) expression, increase ROS production via oxidative stress from NADPH oxidase, and promote pro-fibrotic signaling (e.g., JAK/STAT and NF-κB) in renal epithelial and mesangial cells [78,80]. The mechanistic role of TNF-α has been validated in translational studies with humanized models. Transfer of T cells isolated from hypertensive patients into immunodeficient mice induced elevated blood pressure and renal vascular dysfunction, thus providing evidence of how patient-derived immune cells mediate renal injury in hypertension [81]. The adverse effects were related to increased TNF-α signaling and signals of endothelial draw inflammation. TNF-α is produced by human renal tubular epithelial cells in response to inflammatory signals [82], and it has been established in more recent reviews that TNF-α and its receptors are upregulated by inflammation in human kidney disease, highlighting TNF-α as a central mediator in immune-mediated renal injury [83]. This evidence suggests a reciprocal model whereby TNF-α produced by immune cells inhibits endothelial and epithelial function and, in the injured renal parenchyma, can further potentiate inflammation via autocrine TNF-α signaling, perhaps creating a loop towards sterile inflammation, vascular injury, and fibrosis associated with hypertension-related nephropathy.

Among all of the immune subsets, T helper 17 (Th17) cells have been identified to play a significant role in kidney injury in hypertension. Th17 cells secrete IL-17, a pro-inflammatory cytokine that increases renal sodium reabsorption, vascular inflammation, and fibrosis. Patients with hypertension have elevated IL-17 levels. IL-17A blockade reduces renal inflammation and blood pressure in animal models. Importantly, IL-17A acts together with TNF-α to increase NHE3 and ENaC expression in tubular epithelial cells, both of which promote sodium retention and enhance volume expansion [19,84]. In addition, IL-17A directly induces TGF-β1 and is known to cause interstitial fibrosis and tubular atrophy. In animal models, IL-17-producing γδ T cells are high in the kidneys of hypertensive mice, and both γδ and Th17 cells were identified in human kidney biopsies from hypertensive nephrosclerosis patients [8].

The interaction between the immune system and kidney function creates a self-sustaining feedback loop. Activated T cells infiltrate the renal tissue, altering sodium transport mechanisms and increasing regional oxidative stress. This also recruits additional immune cells and causes renal inflammation. Both innate (e.g., dendritic cells and monocytes) and adaptive (e.g., CD4+ and CD8+ T cells) immune cells participate in this cycle by producing inflammatory mediators and causing fibrosis through mechanisms such as TGF-β and NF-κB activation [85,86]. In the 5/6 nephrectomy model of CKD, sequential transcriptomic analysis has shown a progressive increase in both TLR signaling and inflammasome components [e.g., NLRP3, caspase 1 (CASP1)], which overlap with T-cell infiltration and collagen deposition. This implicates both innate sensors and adaptive effectors in a temporally linked progression of kidney injury [87].

Most significantly, the immune response modulates systemic blood pressure homeostasis. Cytokines such as IL-17, IFN-γ, and TNF-α damage endothelial function and increase vascular stiffness. They downregulate eNOS, impair endothelium-dependent relaxation, and upregulate vascular smooth muscle calcium channels and fibrosis markers. These effects converge to increase vascular resistance and remodeling [78]. Age-related vascular remodeling significantly contributes to renal functional decline in older hypertensive individuals. Structural and functional vascular changes, including increased stiffness, reduced compliance, and glomerular sclerosis, have been observed in aging kidneys and are associated with reduced renal blood flow and filtration rate [88,89,90,91].

Simultaneously, IL-10 and Tregs protect the vasculature by regulating inflammation. Tregs regulate ROS formation, reduce the activation of dendritic cells, and release TGF-β and IL-10, which are the main drivers of preventing T-effector cell activity and endothelial injury. The adoptive transfer of Tregs has been shown to reduce hypertension in mice. Thus, modulation of the ratio between pro-inflammatory and anti-inflammatory immune cells has far-reaching effects on renal injury as well as progressive systemic hypertension [92,93]. Therapeutic strategies aimed at skewing the immune balance, such as IL-17A or CD80 blockade, Treg adoptive therapy, or dendritic cell modulation using isolevuglandin scavengers, represent cutting-edge interventions to blunt hypertensive renal injury at its immunological roots [80].

### 2.4. Mechanical Stress and Podocyte Injury

Podocytes, which comprise the glomerular filtration barrier epithelial cells, are highly sensitive to mechanical stress that occurs with elevated intraglomerular pressure, a phenomenon associated with hypertension. Mechanical stress can cause damaging structural changes, such as podocyte effacement, foot process retraction and detachment, and ultimately, apoptosis. Biomechanical stress activates mechanosensitive ionic channels, including the transient receptor potential cation channel 6 (TRPC6) and Piezo1 channels, to trigger a Ca^2+^ influx coordinating cell signaling processes that lead to cytoskeletal remodeling and injury [94,95]. Piezo1 has emerged as a functional mechanosensor in podocytes, initiating calcium signaling via the nuclear factor of activated T cells, cytoplasmic 1 (NFATc1)/TRPC6 axis, and linking mechanical force to subsequent gene expression and actin disassembly.

Mechanotransduction within podocytes also involves actin cytoskeleton reorganization via molecules such as filamin B, zyxin, and Yes-associated protein (YAP). These are mechanoreceptors that detect mechanical forces by changing the architecture and adhesive properties of podocytes and, thus, their hemodynamic stress resistance. Their overexpression has been observed in both hypertensive and proteinuric kidney disease models [96,97]. YAP/transcriptional co-activator (TAZ) translocate to the nucleus in response to tension, promoting the expression of pro-fibrotic and pro-survival genes, while filamin provides a mechanosensory anchor by linking integrins to the actin network. Zyxin is an LIM-domain protein that modulates focal adhesion dynamics when exposed to mechanical loads, altering podocyte adhesion [98].

These cytoskeletal alterations are intimately linked to proteinuria’s evolution, as structural disassembly injures the filtration barrier. TRPC6 and Piezo1, associated with calcium influx, activate Rho-family GTPases, specifically Rac1, activating lamellipodial extension and actin polymerization. Prolonged Rac1 stabilizes the slit diaphragm components nephrin and podocin. Notably, destabilized slit diaphragm components are associated with detachment and injuries [95]. Calcium signaling by Piezo1 and TRPC6 specifically triggers Ras-related C3 botulinum toxin substrate 1 (Rac1) activation and upregulates injury markers like plasminogen activator inhibitor-1 (PAI-1) and SGK1, exacerbating podocyte stress and glomerular permeability [94,99]. Notably, pharmacological inhibition of Piezo1 with GsMTx4 or inhibition of downstream Rac1 activation significantly inhibited these signaling events, lowered proteinuria, and preserved cytoskeletal architecture in hypertensive and diabetic mouse models [94,100]. In addition, stretched podocytes release microparticles into the urine as early cellular stress biomarkers. This phenomenon has been observed in diabetic and hypertensive models of kidney disease and can potentially act as a diagnostic tool [101]. Researchers are currently investigating these extracellular vesicles (EVs), which contain injury-specific proteins and nucleic acids, as noninvasive urinary biomarkers for podocyte stress, which can detect podocyte stress before clinical proteinuria develops [98].

### 2.5. Endothelial Dysfunction and Hypoxia

Chronic hypertension causes a pathophysiological decrease in renal capillary density, known as capillary rarefaction, which drastically compromises oxygen delivery, resulting in chronic renal hypoxia. This hypoxic environment leads to the stabilization of hypoxia-inducible transcription factors (HIFs), particularly HIF-1α and HIF-2, which regulate the transcription of many genes associated with endothelial cell proliferation and differentiation [via angiogenesis, such as vascular endothelial growth factor (VEGF)], extracellular matrix remodeling, and fibrogenesis [102]. Although this transcriptional response is initially adaptive, chronic activation of HIFs induces maladaptive processes such as epithelial–mesenchymal transition, accumulation of myofibroblasts within the renal interstitium, and ultimately, interstitial fibrosis, thus further contributing to kidney injury [103]. Unlike HIF-1α, endothelial HIF-2α appears to play a protective role. Its deletion leads to increased renal inflammation and fibrosis, indicating that it could be a therapeutic target for maintaining microvascular integrity in hypertensive nephropathy [102]. In contrast, continuous HIF-1α overexpression in tubular epithelial cells can exacerbate EMT due to the upregulation of α-smooth muscle actin and downregulation of E-cadherin (in part via microRNA-21 signaling) [104]. In this inflamed environment, hypoxic endothelial cells upregulate adhesion molecules such as VCAM-1 and ICAM-1, facilitating leukocyte recruitment and perpetuating chronic inflammation [102]. In addition, these immune cells cause tissue injury and promote fibrogenesis.

Another important contributor to hypoxia-induced injury is asymmetric dimethylarginine (ADMA), an endogenous nitric oxide synthase (NOS) inhibitor. ADMA accumulation, most often seen in CKD and hypertension, specifically causes decreased bioavailable NO, impaired vasodilation, increased oxidative stress, and the ability to increase TGF-β1 and collagen deposition as a fibrotic response [105]. Experimental models have shown that ADMA produces glomerular and vascular fibrosis and peritubular capillary rarefaction in a manner mimicking the pathophysiology of human hypertensive nephropathy.

Endothelial dysfunction can contribute to renal tubular injury not only by the description above but also by causing inflammasome activation by the same mechanism, mainly through decreased NO signaling. This was shown in eNOS KO mice, where the absence of endothelial NO resulted in NLRP3 inflammasome activation, more tubular damage, and fibrosis [106].

### 2.6. Metabolic and Energy Dysregulation

Metabolic diseases, such as obesity and diabetes, increase oxidative stress through mechanisms that include advanced glycation end-product (AGE) deposition, activation of protein kinase C (PKC), and dysregulation of the mammalian target of rapamycin (mTOR) signaling pathway. These alterations increase renal fibrogenesis and endothelial injury in patients with hypertension [107]. AGEs target their binding receptor RAGE on endothelial and tubular epithelial cells and induce the activation of NADPH oxidase, which produces ROS and releases pro-inflammatory cytokines (IL-1β, TGF-β1), causing further kidney injury in a state of hypertension [108]. Apart from systemic determinants, metabolic dysfunction in kidney cells is recognized as an important factor leading to hypertensive kidney injury. All renal tubular epithelial cells face continued stress (high salt, pressure, glucose, or Ang II), with metabolic reprogramming of the normal stress response towards anxiety/glycolysis, induced by the Warburg-like effect associated with cancer metabolism. Reprogramming is maladaptive in the kidneys, where cellular behavior is energy-demanding and fatty acid oxidation (FAO) is the primary source of ATP needed to sustain renal function [109]. In particular, stress-activated renal tubular cells display defective glycolytic enzyme function, such as hexokinase II, and an increased preference for energy substrates toward inefficient glycolysis rather than fatty acid oxidation. These changes impair cellular ATP production and introduce energy stress, leading to deficient nephron function and promoting CKD progression [110,111]. Loss of peroxisome proliferator-activated receptor alpha (PPARα), a crucial regulator of FAO, and impaired activity of carnitine palmitoyltransferase-1 (CPT1) have been associated with increased lipid accumulation, swelling of mitochondria, and higher apoptosis in the tubular segments of hypertensive mice [112].

Disruption of the mTOR pathway is a major driver of energy deficits. Early phases of hypertension-induced renal injury have been seen to inhibit mTORC1 while concurrently activating AMP-activated protein kinase (AMPK), indicative of a compensatory mechanism in response to low NADH and ATP levels. This transitional dynamic aims to sustain energy homeostasis; however, chronic AMPK activation, continuous mTORC1 inhibition, or a combination of both causes reorganized cytoskeletal structure, lower protein synthesis, increased autophagy, and tubulointerstitial remodeling [113].

Moreover, damaged mitochondrial metabolism can cause the accumulation of acylcarnitines and ROS, which disrupt the mitochondrial membrane, leading to a pathogenic assemblage of oxidative stress, followed by ATP depletion. These changes disrupt nephron-segment-specific functions like sodium reabsorption and solute handling, thus hastening the disconnection of the glomerular–tubular units and fibrosis [114,115]. In addition, defective fatty acid oxidation and glucose toxicity exacerbate mitochondrial damage and oxidative stress. Oxidative stress not only damages proteins and DNA but also triggers immune activation, inflammation, and activation of fibrotic signaling cascades such as TGF-β, extending kidney injury [116,117]. Mitochondrial ROS activate the nuclear factor-kappa B (NF-κB) and NLRP3 inflammasome pathways, activating the release of interleukin-1β and recruiting macrophages into the renal interstitium, promoting the fibrotic process [118].

### 2.7. Epigenetic and MicroRNA Regulation

In addition to genetic susceptibility, epigenetic modifications, such as DNA methylation, histone acetylation, and microRNA (miRNA) regulation, play critical roles in the pathogenesis of hypertension-induced kidney injury. These heritable yet reversible modifications influence chromatin accessibility and post-transcriptional gene regulation, thereby modulating fibrosis, inflammation, and oxidative stress pathways [119].

**Figure 4:**
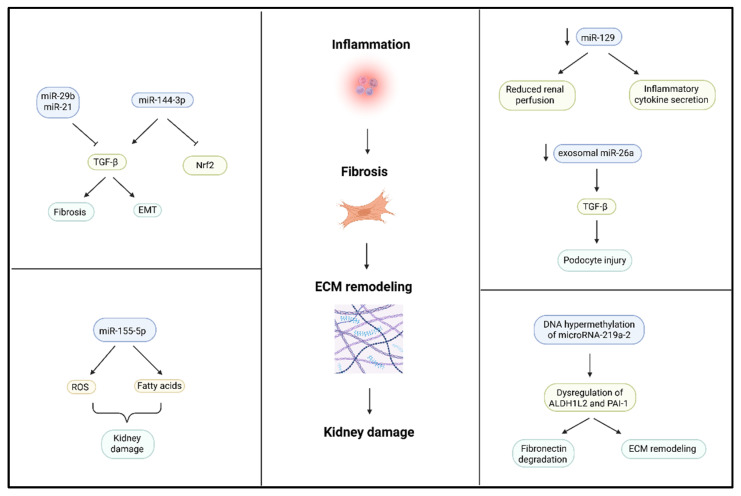
MicroRNA-directed regulation of important molecular pathways driving hypertensive kidney injury: This diagram depicts how particular microRNAs alter the signaling cascades involved in inflammation, fibrosis, and extracellular matrix (ECM) remodeling in the pathophysiology underlying hypertensive nephropathy. Each of the four quadrants illustrates different pathways based on microRNAs. The top left panel indicates that the pro-fibrotic miR-29b and miR-21 upregulate transforming growth factor-beta (TGF-β) signaling, which can provoke fibrosis and epithelial–mesenchymal transition (EMT), and miR-144-3p suppresses the antioxidant regulator nuclear factor erythroid 2-related factor 2 (Nrf2), which provokes fibrotic signals. The top right panel shows that downregulation of miR-129 reduces renal blood flow and stimulates the secretion of inflammatory cytokines. Inhibition of exosomal miR-26a results in increased TGF-β-mediated podocyte injury. The bottom left panel shows that the pro-inflammatory miR-155-5p increases reactive oxygen species (ROS) and dysregulates fatty acid metabolism, contributing to renal parenchymal injury. Finally, the lower right panel illustrates that hypermethylation of microRNA-219a-2 contributes to the downregulation of aldehyde dehydrogenase 1 family member L2 (ALDH1L2) and plasminogen activator inhibitor-1 (PAI-1), which degrades fibronectin and enhances extracellular matrix (ECM) remodeling. All of the changes in these microRNAs can initiate the cascade of inflammatory responses, fibrosis, and disorganized ECM repair processes that promote irreversible injury.

MiRNAs have the potential to function as gene expression regulators in renal fibrosis, inflammation, and oxidative stress. Dysregulation of microRNAs (miR-144-3p and miR-129) has been shown to enhance pro-fibrotic mediator expression (TGF-β) and suppress antioxidant defense pathways (Nrf2), thereby causing inflammation, oxidative stress, and renal tissue fibrosis (Figure 4) [120]. In animal models of Ang II-induced hypertension, altered renal microRNA expression is linked to loss of function. A reduction in miR-129, for instance, correlates with reduced renal perfusion and enhanced inflammatory cytokine secretion but is reversed upon therapy, such as administration of hydrogen sulfide (H_2_S), correcting the miRNA profile and kidney function [121]. In addition to individual miRNAs, more widespread epigenetic changes, such as DNA hypermethylation of microRNA genes, can also play a role in renal fibrosis. Hypermethylation of microRNA-219a-2, for instance, represses its expression, with secondary target dysregulation of targets such as aldehyde dehydrogenase 1 family member L2 (ALDH1L2) and plasminogen activator inhibitor-1 (PAI-1), which are pivotal in fibronectin degradation and extracellular matrix remodeling [62]. Certain miRNAs have renoprotective activity and suppress fibrotic signaling pathways. An example is miR-29b, which inhibits TGF-β-induced epithelial–mesenchymal transition (EMT) and fibrosis in hypertensive rats and, thus, is a promising drug candidate [122]. Other studies have identified miR-155-5p, which is elevated in mice with salt-sensitive hypertension and is responsible for renal damage by ROS generation and dysregulation of fatty acid metabolism [123]. Additionally, some recent studies have identified urinary and exosomal miRNAs as noninvasive biomarkers of chronic kidney hypertension. Riffo-Campos et al. (2022) evaluated exosomal miR-26a and showed that it was downregulated in patients with albuminuria and contributed to podocyte injury through the TGF-β signaling pathway, and its capability as a diagnostic agent was reinforced [124]. Similarly, miR-21 has also been shown to be associated with renal fibrosis and is observed prior to albuminuria, indicating that it is an indicator of kidney injury earlier in the disease [125]. Circulating miRNAs, such as let-7g-5p and miR-191-5p, can predict CKD in hypertensive patients [126]. Finally, Petzuch et al. (2022) discovered that urinary miRNA profiles from EVs increased sensitivity to the potential for fibrotic and glomerular injury due to miRNA changes, reaffirming the clinical relevance of EV enrichment in miRNA assays [127].

Pharmacoepigenetics is also crucial. Some antihypertensive agents (e.g., candesartan) have an effect on epigenetic status, either by modifying miRNA profiles or by altering DNA methylation; hence, they possibly possess the twofold benefit of blood pressure management and prevention of renal injury. Together, epigenetic mechanisms and miRNA networks form a regulatory axis that determines susceptibility to renal fibrosis, oxidative stress, and inflammation in hypertensive individuals. Intervention on these epigenetic regulators is a potential path toward novel diagnostics and therapeutics for hypertensive nephropathy [128].

### 2.8. Vascular Smooth Muscle and Cytoskeletal Dynamics

Altered function and phenotypic plasticity of vascular smooth muscle cells (VSMCs) are increasingly recognized as central contributors to hypertension-induced renal injury. Under hypertensive stress and exposure to mediators such as Ang II and uric acid, VSMCs undergo phenotypic switching from the contractile to synthetic state. This remodeling is characterized by augmented cell proliferation, migration, and extracellular matrix deposition, and it is linked to reorganization of cytoskeletal proteins, such as α-smooth muscle actin (α-SMA), smoothelin, and SM22α. This phenotypic transition leads to vascular stiffening and reduced compliance, contributing to increased renal vascular resistance, altered glomerular hemodynamics, and subsequent glomerular injury [129].

Mechanotransduction plays a crucial role in these processes. Mechanical stimulation, which is elevated intravascular pressure, stimulates signaling pathways such as Ras homolog family member A/Rho-associated coiled-coil containing protein kinase [RhoA/Rho-associated coiled-coil containing protein kinase (ROCK)] [130], extracellular signal-regulated kinase ½ (ERK1/2) [131], and the phosphoinositide 3-kinase/protein kinase B (PI3K/AKT) pathway [132], which regulate cytoskeletal remodeling and VSMC contraction. These pathways also mediate the nuclear translocation of transcription factors such as myocardin-related transcription factor-A (MRTF-A), along with serum response factor (SRF), inducing the expression of smooth muscle master contractile genes and bolstering VSMC phenotypic programming [133]. RhoA/ROCK signaling promotes actin polymerization and stress fiber formation, and continuous RhoA/ROCK activation promotes synthetic VSMC activity, increasing collagen I/III production and pro-fibrotic cytokine release (e.g., TGF-β1). The ERK1/2 and c-Jun N-terminal kinase (JNK) pathways support VSMC proliferation, whereas the PI3K/AKT pathway influences cell survival, matrix production, and the inflammatory response [134].

Interruption of actin filament integrity or regulatory proteins, such as γ-adducin, attenuates the myogenic response and renal blood flow autoregulation and augments hypertensive damage to the kidneys [135,136]. In experimental models, the lack of γ-adducin leads to diminished VSMC cytoskeletal integrity, impaired mechanotransduction, and disturbed renal autoregulation, indicating the importance of actin integrity in vascular remodeling under the stress of hypertension [130].

In addition, the transcription factors fragile X mental retardation syndrome-related protein 1 (FXR1) and tonicity-responsive enhancer binding protein/nuclear factor of activated T-cells 5 (TonEBP/NFAT5) link actin polymerization and cytoskeletal gene expression to mechanical stress. FXR1 stabilizes mRNAs for important cytoskeletal proteins, including ACTA2 and calponin, and thus helps maintain VSMC contractility and vascular tone. TonEBP/NFAT5 is conditioned by osmotic and mechanical loads and modifies cytoskeletal remodeling and the expression of inflammatory genes in hypertensive vessels. Reduced FXR1 lowers mRNA stability in cytoskeletal proteins and reduces the activity of VSMC contraction, adhesion, and blood pressure regulation. Hypertensive pathological arterial remodeling has a dominant mechanosensitive component, in which chronic cyclic strain and pressure lead to structural rearrangement of the actin cytoskeleton and VSMC dedifferentiation, perpetuating vascular and renal injury as a feedforward loop. Targeting mechanotransductive signaling, or restoring cytoskeletal homeostasis, may be a therapeutic option in hypertensive nephropathy [137,138].

## 3. Immune Mechanisms Underpinning Renal Injury

### 3.1. T-Cell Activation and Cytokine Responses

T-cell infiltration and activation, particularly of the CD4+ and CD8+ subsets, in renal tissue are key events in the pathogenesis of hypertension-induced kidney injury. These lymphocytes are recruited early in response to endothelial dysfunction, increased shear stress, and oxidative signals, and they accumulate in the renal cortex and perivascular sites, where they amplify inflammation and fibrotic changes [139]. These inflammatory cells produce pro-inflammatory cytokines such as IL-6, TNF-α, and IL-17, which have a direct causative effect on inflammation, oxidative stress, and fibrosis. IL-17, released by Th17 and γδ T cells, increases renal sodium reabsorption by promoting chemokine (e.g., MCP-1) and adhesion molecule expression, as well as neutrophil recruitment. In addition, TNF-α and IL-6 promote fibroblast activation and matrix deposition [140]. The cytokine cascade increases structural injury to the kidneys, aggravates function, and induces the progression of chronic kidney disease in the hypertensive population [80,141].

Experimental models have shown that blocking T-cell activation, especially via co-stimulatory pathways (e.g., CD28-CD80/CD86 axis), significantly reduces renal inflammation and fibrosis in the kidneys. Inhibition of CD28 signaling interrupts the second signal necessary for naïve T-cell activation and has been shown to inhibit T-cell expansion and renal infiltration in Ang II-infused mice [139]. Agents that neutralize IL-17, inhibit Janus kinase-signal transducer and activator of transcription (JAK-STAT) signaling, or suppress CD4+ T-helper cell polarization exhibit renoprotective effects even without major reductions in blood pressure [142,143]. Ang II is the pivotal molecule to link hypertension with immune activation by direct stimulation of T-cell cytokine release and tilting the balance towards pro-inflammatory Th1/Th17 phenotypes at the expense of regulatory T cells (Tregs). Ang II signals through AT1R on antigen-presenting cells (APCs) and T lymphocytes, increasing the production of IL-6 and IL-23, which drives Th17 expansion and suppresses IL-10-producing Tregs [139]. This immune shift aggravates fibrosis and increases glomerulosclerosis, while immune modulation strategies like mycophenolate mofetil, which suppresses T- and B-lymphocyte proliferation via inosine monophosphate dehydrogenase inhibition, have been shown to reduce proteinuria, T-cell infiltration, and renal collagen deposition in hypertensive nephropathy models [144].

### 3.2. Role of the NLRP3 Inflammasome

The NLRP3 inflammasome is a key innate immune molecule that plays a central role in the pathogenesis of hypertensive renal injury. It is a cytoplasmic danger sensor that recognizes stress stimuli, such as ROS, ATP, and ionic flux, and induces the processing and secretion of pro-inflammatory cytokines, specifically IL-1β and IL-18, which promote inflammation and are responsible for renal tissue fibrosis [145]. Experiments have revealed that NLRP3 activation in immune cells (macrophages and dendritic cells) and renal resident cells, such as the tubular epithelium, is prevalent and is responsible for acute [146,147,148] and chronic kidney injury [145,149,150], including hypertensive nephropathy [9,151,152], through both canonical inflammasome activation and non-canonical pathways of mitochondrial stress and pyroptosis [153]. New therapies directed toward the NLRP3 inflammasome are increasingly being recognized for their potential to decrease both AKI and CKD, such as hypertensive nephropathy. Studies using inhibitors such as MCC950 have proven effective in human and animal models because of their ability to inhibit NLRP3 inflammasome-mediated activation of IL-1β and IL-18, which can reduce renal inflammation, fibrosis, and pyroptosis. In studies of MCC950, the inhibitor modestly lowered blood pressure and kidney dysfunction in salt-sensitive hypertension [154] and renal fibrosis following cisplatin treatment, where decreased oxidative stress and inflammation signaling were observed [155]. Inhibitors that selectively target NLRP3, such as CY-09, have demonstrated renoprotective characteristics in preclinical models of diabetic nephropathy [156] by blocking downstream activation of cytokines through inhibition of inflammasome activation and cytokine release. Although these therapies are currently only in the experimental or phase I/II clinical trial stages, the benefits observed in preclinical studies support their translational value in humans. These types of therapies indicate the urgency of providing these targeted therapies, and that NLRP3 inhibition may represent a new therapeutic intervention in cases of renal inflammatory and fibrotic diseases related to hypertensive and other kidney conditions [157,158].

### 3.3. Neuroimmune Interactions

Recent studies have illuminated the complex bidirectional interactions between the sympathetic nervous system (SNS) and the immune system in hypertensive kidney injury. Sympathetic elevation, which is common in CKD and essential hypertension, serves as a neurohumoral exacerbator of renal inflammation. Catecholamines with pro-inflammatory properties, including norepinephrine, bind to β_2_-adrenoreceptors or α_1_-adrenoreceptors on immune cells, including T cells and macrophages, and actively promote the release of IL-6, TNF-α, and IFN-γ and adopt a pro-inflammatory Th1/Th17 phenotype. This sympathetic drive not only exacerbates cytokine-mediated renal injury but also induces a prominent pattern of upregulation of major histocompatibility complex (MHC) class II molecules on antigen-presenting cells that supports the maintenance of adaptive immune responses. Furthermore, sympathetic innervation of the spleen is an essential route for central–peripheral immune communication, and splenic nerve ablation has been demonstrated to blunt hypertension in experimental hypertensive models [33]. The kidney itself is both a generator and target of sympathetic activity. Afferent sensory nerves in the damaged kidneys deliver excitatory inputs to the central sympathetic centers, reinforcing the overactivation loop. Evidence from patients with ESKD shows that bilateral nephrectomy normalizes sympathetic activity, further confirming the kidneys’ role in perpetuating neuroimmune dysregulation [159]. Interventions directed toward this neuroimmune interface, such as RDN, have been shown to lower blood pressure and to reduce immune-mediated inflammation in the kidneys. For instance, in hypertensive obese rat models, RDN reduces kidney inflammation, albuminuria, and renal fibrosis, independent of systemic metabolic gain [160]. In addition, sympathetic blockade affects the GFR via hemodynamic mechanisms. Sympathetic stimulation attenuates renal blood flow by causing afferent arteriolar constriction, whereas sympathetic blockade causes afferent arteriolar dilation. However, this may not always increase the GFR, because changes in renal perfusion pressure may decrease glomerular capillary pressure and, thus, decrease the GFR [161]. In addition, sympathetic activation modulates the autoregulatory threshold for renal blood flow and GFR, allowing the kidney to be even more prone to hypofiltration once sympathetic tone is lifted [162,163].

## 4. Genetic Susceptibility and Epigenetic Regulation

### 4.1. Genetic Variants and Renal Hemodynamics

Animal model studies have identified several genetic variants that significantly affect renal autoregulation and susceptibility to hypertensive kidney injury. A critical mechanism is the myogenic response of the afferent arteriole, which protects the glomerular capillaries from excessive systemic pressure fluctuations. This autoregulatory reflex is vital for maintaining constant glomerular filtration under varying blood pressures and involves rapid depolarization, calcium influx, and smooth muscle contraction in response to the stretch. In models highly susceptible to kidney injury in Fawn-Hooded Hypertensive (FHH) and Dahl Salt-Sensitive (SS) rats, this myogenic mechanism is impaired, leading to inadequate afferent arteriole constriction, glomerular hypertension, and consequent structural injury, including mesangial expansion, capillary collapse, and albuminuria [164].

Various genetic loci and candidate genes have been implicated in maladaptive responses to stress. One of these is ADD3 (γ-adducin), which encodes a cytoskeletal scaffolding protein responsible for actin filament capping and membrane–cytoskeleton interactions. ADD3 mutations disrupt afferent arteriole contractility by interfering with the actin cytoskeletal architecture, resulting in a loss of myogenic tone and increased glomerular capillary pressure, ultimately resulting in proteinuria and glomerulosclerosis in FHH rats. [135]. Another important regulator is DUSP5, a dual-specific phosphatase that inactivates ERK1/2 in vascular smooth muscle cells. In SS rats, loss-of-function variants in DUSP5 amplify MAPK signaling and diminish the vasoreactivity of afferent arterioles, thus impairing renal autoregulation under salt loading or Ang II infusion, causing greater glomerular strain and worsening kidney injury [165]. Arhgef11, a Rho guanine nucleotide exchange factor, is another important modulator of vascular tone and cytoskeletal dynamics. In SS rats, Arhgef11 overexpression increases RhoA-ROCK signaling, promotes actomyosin contractility, and activates fibrotic pathways that promote epithelial–mesenchymal transition (EMT), eventually increasing renal collagen deposition and causing structural degeneration of the kidney. Knockout of Arhgef11 or inhibition with ROCK inhibitors like fasudil restores afferent arteriole tone changes, decreases intraglomerular pressure, reduces proteinuria, and limits the degree of fibrotic transformation in models of salt-sensitive hypertension [166,167].

While these results were derived from animal models, human data reinforce the genetic susceptibility to hypertensive kidney injury. A pivotal study conducted by Parsa et al. used *APOL1* risk alleles to examine the importance of genetic risk in African-American patients with hypertension-associated CKD. Individuals with two risk variants (G1 or G2) had greater rates of decline in kidney function and over two times the risk of kidney failure, independent of blood pressure. These data sufficiently implicate all *APOL1* variants in glomerular injury and podocyte dysfunction and connect genetic risk to the progression of kidney disease [168]. In parallel, advances in omics technologies have facilitated the identification of novel transcriptomic and proteomic signatures that influence vascular tone, sodium handling, and renal remodeling in hypertension. Integrating multi-omics data enables the discovery of patient-specific molecular pathways contributing to hypertensive and resistant hypertension phenotypes [169]. Building upon this, the Kidney Precision Medicine Project provides a comprehensive framework for incorporating multidimensional data, including kidney tissue transcriptomics, histopathology, and clinical phenotypes, to refine our understanding of CKD’s heterogeneity. These integrated data layers can enhance the classification of hypertensive nephropathy and guide individualized interventions based on molecular mechanisms [170].

### 4.2. The Role of P66SHC and Cytoskeletal Regulators

Recent evidence has highlighted that adaptor protein p66Shc plays a vital role in mediating renal vascular dysfunction in hypertension; p66Shc, with its gene *Shc1,* is overexpressed in salt-sensitive hypertension rats and is involved in impaired myogenic responsiveness of renal preglomerular arterioles. By inhibiting the activation of transient receptor potential (TRP) channels in VSMCs, p66Shc inhibits calcium signaling, decreasing cytosolic Ca^2+^ influx and the contractile response needed for pressure autoregulation [20]. Meanwhile, p66, a Shinya deletion, normalizes vascular tone and glomerular defense against hypertension-induced nephropathy, as evidenced by knockout animals. The protein also regulates dynamic calcium oscillations in renal vascular smooth muscle via interaction with endothelin-1 signaling, which modulates vascular stiffness and perfusion pressure [171]. From a mechanotransduction perspective, cytoskeletal regulators such as actin-associated proteins are also disrupted in the presence of excess p66Shc activity. In nephrotoxicity models, p66Shc is associated with heat shock protein 27 (Hsp27), the principal actin cytoskeleton stabilizer, promoting accelerated disorganization and apoptosis during stressful conditions, and also sensitizing the kidneys to hypertension-induced damage [172].

## 5. Targeted Therapeutics

Numerous precision medicine strategies have been investigated to target the molecular heterogeneity of hypertensive renal injury. These include genetically guided therapy, biomarker-based treatment regimens, and systems biology tools allowing for the stratification of patients based on molecular signatures. An overview of these strategies is provided in Table 2.

### 5.1. Genetically Guided Pharmacotherapy

Genetically guided pharmacotherapy represents a new dimension in precision medicine that aims to optimize the treatment of hypertension based on an individual’s personal genetic data. This practice considers polymorphisms in genes affecting blood pressure control and response to drugs to create individualized therapies with improved efficacy and reduced side effects. One example is a variant in the lysine-specific demethylase-1 (*LSD1*, *KDM1A*) gene, which is particularly relevant in salt-sensitive hypertension, which is more prominent in individuals of African ancestry. Carriers of *LSD1* polymorphisms have a lower response to most first-line antihypertensive agents, such as calcium channel blockers, and a better response to MR antagonists, such as eplerenone or spironolactone. In a recent clinical trial, these patients said that they had a better blood pressure reduction from MR antagonists than from amlodipine [177]. In addition to *LSD1*, several pharmacogenetically relevant polymorphisms have been introduced. For example, the ADRB1 Arg389Gly variant alters β-blocker response, whereby Gly389 carriers have greater responsiveness to drugs (metoprolol) [178]. Polymorphisms in the *CYP2D6* and *CYP3A5* genes, for example, can alter the metabolism of metoprolol and amlodipine, respectively, and influence optimal dosing regimens [179,180]. Another well-studied locus is the angiotensin-converting enzyme (ACE) gene polymorphism. Polymorphism in ACE (I/D) contributes to inter-personal variability in plasma ACE activity and may play a role in determining the response to ACE inhibitors; however, its clinical applicability is population-dependent [181].

Multi-omics and genome-wide association studies (GWASs) have identified hundreds of blood pressure-associated variants, many of which influence renal gene expression and the metabolism of drugs. Specifically, Eales et al. identified more than 170 kidney-expressed genes that are presumably involved in the pathogenesis of hypertension. These genes, such as *SLC12A3* (encoding the thiazide-sensitive sodium chloride cotransporter), *NOS3* (endothelial nitric oxide synthase), and *SH2B3* (an immune regulator), are linked to known drug targets, creating a window of opportunity for genetically informed repurposing and optimization of existing therapies [182].

Pharmacogenomic panel testing with more than one gene variant is also gaining popularity. Simulation models suggest that this could improve blood pressure control, reduce side effects, and lower healthcare costs. Kelley et al. showed a 47% cost reduction over three years in a 10-million-patient simulation, primarily by preventing ineffective or dangerous drug choices due to genetic mismatch [183].

Moreover, gene therapy has been investigated to restore the molecular pathogenesis of hypertensive heart disease. Although not yet implemented clinically, many studies have shown that gene transfer to the RAAS, NO signaling, or sodium-handling pathways normalizes blood pressure and prevents kidney injury in models of hypertension. For example, adenovirus-mediated transfer of ACE2, AT2R, or eNOS in rodent models showed favorable antihypertensive and nephroprotective properties. Similarly, knockdown of Na^+^/H^+^ exchanger isoforms (i.e., NHE3) decreased salt-sensitive hypertension in preclinical trials [184,185].

While pharmacogenomic profiling is now becoming more familiar within personalized medicine, the majority of gene therapeutic approaches for hypertension have been stuck in preclinical animal studies, with attempts to advance them to human clinical trials failing many times. Historical experiments focused on gene therapy to alter the RAAS. Gene transfer of ACE2 through adeno-associated virus (AAV) vectors was able to lower blood pressure and ameliorate cardiac hypertrophy in hypertensive rodents [186]. Another promising strategy of silencing angiotensin II type 1 receptor (AT1R) using RNA interference showed beneficial hemodynamic properties in animal models [187]. Little progress toward routine clinical application has been achieved in the 40 years since the first gene therapy product for severe immunodeficient disorders (Strimvelis, GSK), despite continued work to optimize vector safety, tissue-specific targeting, immune responses to gene therapy, and durable transgene expression in treatment-resistant hypertension. It is important to highlight that hypertension is a complex trait that is polygenic and influenced by the environment. Therefore, single-gene targeting may be inadequate. Studies employing clustered regularly interspaced short palindromic repeats (CRISPR)/CRISPR-associated protein 9 (Cas9)-based gene editing technologies continue to expand opportunities in monogenic forms of hypertension, including Liddle syndrome and familial hyperaldosteronism [188].

### 5.2. Device-Based Interventions

Device treatments, particularly catheter-based renal denervation (RDN), have proven to be effective strategies for the management of treatment-resistant hypertension and prevention of associated kidney injury. RDN blocks the efferent and afferent renal sympathetic nerve activity, which is typically overactive in hypertensive patients and leads to both systemic blood pressure elevation and chronic kidney damage. Fresh devices, such as the Netrod™ six-electrode system and ultrasound systems, offer targeted ablation of renal nerves with greater safety and longevity [189]. Multi-electrode radiofrequency systems, such as the Symplicity Spyral and Vessix catheters, facilitate circumferential and effective renal nerve ablation. Clinical trials such as SPYRAL HTN-ON MED and REDUCE-HTN have demonstrated that RDN can produce clinically significant ambulatory and office blood pressure reductions with a favorable safety profile [174,190]. In a recent sham-controlled trial in Chinese patients with uncontrolled hypertension on triple therapy, RDN with a self-adaptive six-electrode system reduced 24 h systolic blood pressure by −9.4 mmHg, significantly better than sham procedures, with minimal complications [174]. Moreover, economic modeling in the UK predicted that RDN would be cost-effective, with an incremental cost-effectiveness ratio (ICER) of GBP 13,482 per QALY gained, well within the NICE thresholds [191].

Although RDN was originally designed to reduce blood pressure, its benefit also translates to renal protection. Preclinical and clinical evidence indicates that RDN reduces renal oxidative stress, inflammation, and fibrosis by suppressing sympathetic overactivity. These effects are even observed in models where reducing blood pressure is not the primary cause of kidney protection, suggesting a direct renal benefit from sympathetic modulation [192,193]. Recent studies show that RDN also controls renal microRNA expression, enhances microvascular perfusion, and reduces tubulointerstitial fibrosis and glomerular injury, with long-term renoprotective effects. These findings suggest that aside from blood pressure control, RDN may be implicated in the retardation or prevention of CKD progression in hypertensive patients [194,195].

Pathophysiologically, RDN not only decreases systemic sympathetic tone but can also be implicated in renal protection, opposing the evolution of hypertensive nephropathy by correcting renal hemodynamics and reducing glomerular hyperfiltration [196]. These encouraging results are leading to a renewed clinical interest in RDN, now supported by updated European guidelines and real-world experience that both attest efficacy and safety in resistant and uncontrolled hypertension patients [197].

### 5.3. Targeting Inflammatory and Immune Pathways

The central involvement of inflammation and immune dysregulation in hypertensive renal damage makes such pathways promising candidates for precision medicine therapy. Cytokines, such as IL-17, and immune cells, such as Th17 lymphocytes, are significant mediators of kidney damage, sodium retention, fibrosis, and vascular remodeling. IL-17 blockade with monoclonal antibodies has also been shown in preclinical models to reduce renal inflammation and blood pressure in hypertensive animal models [8,19]. Several cytokines in the Th17 family, including IL-17F and IL-22, have also been implicated in glomerular inflammation and immune cell recruitment in the kidneys, promoting further exacerbation of tissue injury in models of glomerulonephritis [198]. Other immunomodulatory approaches are being investigated to restore the Th17/Treg balance, inhibit chemokine-induced leukocyte migration, and inhibit hyperactivation of the innate immune system. The IL-6-gp130-STAT3 pathway is critically important for Th17 differentiation, and knockout of this pathway could result in suppression of IL-17-driven inflammation without broadly blunting immune function [199]. Therapeutics targeting IL-1, which drives Th17 differentiation and inflammation via glycolysis, may also provide therapeutic benefits in autoimmune models that could be translated in the context of renal inflammation [200]. Both clinical and experimental evidence suggests that modulation of immune networks (as opposed to individual cytokines) provides stronger and more sustained control of hypertension-driven inflammation [201,202]. New methods of regulating renal inflammation without harming systemic immune competence, such as complement inhibition and vagus nerve stem stimulation, are emerging. C3a, a complement component, has been shown to promote IL-17 production in T cells and renal fibrosis in experimental studies [203,204,205]. These therapies may be optimally used when combined with traditional antihypertensives, such as RAS inhibitors, with the additive effects of blood pressure reduction and renal protection.

### 5.4. Metabolic Modulators and mTOR Inhibition

An increasing amount of research has recognized the pivotal role of the mTOR signaling pathway in the development and progression of hypertensive kidney injury. In the state of metabolic overload, such as salt-sensitive hypertension, mTOR (and especially mTORC1) is overactivated, resulting in suppressed autophagy, exacerbated oxidative stress, and accelerated renal fibrosis [206]. Pharmacological mTOR inhibition with agents such as rapamycin has been shown to restore autophagic flux, reduce renal inflammation, and protect against kidney damage caused by excessive salt intake in experimental models [207]. There may be a synergistic effect demonstrated in synthetic non-coding oligonucleotides targeting mTOR and STAT3, exhibiting anti-fibrotic and anti-inflammatory effects via bioactives in obstructive nephropathy [208]. In addition, there are a range of other treatments focused on improving autophagy via indirect mTOR pathway modulation by the PI3K/Akt/mTOR pathway, or via activation of AMPK, which also shows renal protection [209]. Kaempferol reduces apoptosis and promotes autophagy via the AMPK/mTOR pathway in diabetic nephropathy models [210]. While there is a physiological rationale and preclinical evidence for mTOR inhibitors in hypertensive kidney disease, there are no registered clinical trials with a focus in this population. Additionally, there are safety concerns, including dyslipidemia and immunosuppression. However, continued advancement of selective mTOR inhibitors may lessen most of these concerns. Thus, there is translational promise for mTOR inhibition in hypertensive kidney disease, but further studies in patient populations using rigorously designed studies should be sought.

The therapeutic value of mTOR modulators is not limited to CKD treatment. In AKI, CaMKIV-mTOR-dependent autophagy is cytoprotective and important for regulating inflammation [211]. The dual effects of mTOR, promoting and inhibiting autophagy depending on other cellular conditions, underscore the complexity of its regulation and the need for precision in any intervention. Combining mTOR inhibitors or metabolic enhancers with other specific agents in combination therapy now represents a larger opportunity to direct treatment more effectively and manage the inflammatory and metabolic processes associated with the development of hypertensive kidney disease.

### 5.5. Personalized Therapeutic Combinations

Precision medicine for hypertensive kidney disease is rapidly moving toward rational combination therapy, targeting several pathogenic mechanisms simultaneously. While blood pressure control is the foundation, pairing agents targeting hemodynamic, metabolic, inflammatory, and fibrotic pathways may provide synergistic protection to the kidneys. An excellent example is the use of RAS inhibitors (such as ACE inhibitors or ARBs) in combination with SGLT2 inhibitors, which not only improves glycemic control but also decreases intraglomerular pressure and oxidative stress. A meta-analysis of 10 RCTs involving approximately 17,000 patients revealed that the addition of SGLT2 inhibitors to RAS blockers significantly reduced cardiovascular death, end-stage renal disease progression, and albuminuria compared to RAS blockers alone [176]. Triple therapy also yields greater gains, as demonstrated in preclinical models of Alport syndrome. RAS inhibitors, SGLT2 inhibitors, and finerenone (non-steroidal mineralocorticoid receptor antagonist) were administered to mice, and in comparison with two-drug therapies they showed significant survival prolongation and minimal interstitial fibrosis [212]. This treatment approach is also supported by clinical experience. Patients with rapidly progressive diabetic nephropathy experienced substantial reductions in proteinuria and stabilization of eGFR when treated with a combination of RAS blockade, SGLT2 inhibitors, and glucagon-like peptide-1 (GLP-1) receptor agonists [213]. Furthermore, biomarkers such as endotrophin (ETP), a marker of fibrosis, are reduced more optimally by dual RAS/SGLT2 blockade than by monotherapy, offering promising prospects for monitoring treatment response [214]. These findings indicate that multi-targeted, biomarker-guided regimens may become the standard treatment for progressive hypertensive or diabetic kidney disease in the near future.

Clinical evidence for personalized combination therapy arises from diabetes and diabetic nephropathy, although clinical trials focusing on hypertensive nephropathy are underway. The EMPA-KIDNEY trial evaluated the SGLT2 inhibitor empagliflozin in patients with CKD of mixed etiology, including patients with primary hypertension. There was a clear effect on renal protection and the risk of cardiovascular events in non-diabetic hypertensive patients, providing justification for SGLT2 inhibitors in the context of hypertensive kidney disease management [215]. The combined analysis of the evidence for finerenone in the FIDELITY pooled analysis of the FIDELIO-DKD and FIGARO-DKD studies showed that finerenone slowed the progression of CKD in the presence of diabetes but also considerably improved blood pressure control and markers of inflammation, suggesting that this agent may be worth considering in clinical practice for combination therapy in hypertensive kidney disease [216]. In comparison, combinations containing glucagon-like peptide-1 (GLP-1) receptor agonists (e.g., liraglutide or semaglutide) have mostly been studied in diabetic populations, and limited evidence directly supports their use in non-diabetic hypertension-related CKD. Likewise, limited evidence exists recommending the clinical use of dipeptidyl peptidase-4 (DPP-4) inhibitors and endothelin receptor antagonists in the treatment of hypertensive nephropathy, although there is good evidence in diabetic kidney disease. Consequently, while some regimens, such as RAAS + SGLT2 inhibitors, have evidence of benefit in CKD with different causes (which included hypertension), combinations with GLP-1RAs or DPP-4 inhibitors need to be evaluated further in hypertensive CKD.

Peptide-based interventions are becoming prominent precision medicine strategies, in part because of their specificity, low toxicity, and multifunctional regulatory effects, in addition to genetic and molecular precision. Peptide 17 downregulated the Hippo/YAP signaling pathway, reducing inflammation and fibrosis in the kidney tissue, and ultimately reducing the extent of early hypertension-related renal injury [217]. Furthermore, specific soybean-based antihypertensive peptides can mitigate angiotensin II-induced renal damage by modulating the MAPK and NF-κB signaling pathways, highlighting their anti-inflammatory and antioxidative effects [218]. In addition to the great potential of peptide therapeutics in precisely mitigating hypertension-induced renal damage, peptide classifiers, such as CKD273 and specific custom urinary peptide panels, may also fulfill early diagnostic and therapeutic lead potential [219]. These observations strongly confirm the ability of peptide therapeutics to contribute to multimodal dimensionality and expand the leverage of precision medicine strategies integrated into the broader consideration set of hypertension-related kidney injury.

### 5.6. Pediatric Precision Approaches

Recent studies emphasize the growing importance of precision medicine in managing pediatric hypertension, a condition increasingly linked to long-term renal and cardiovascular damage. Central to this approach is the early identification of biomarkers, particularly components of the RAAS, which can direct more targeted and effective interventions. The Pediatric Hypertension and Renin–Angiotensin SystEm (PHRASE) study emphasized the clinical value of angiotensin-(1-7) and angiotensin-converting enzyme 2 (ACE2) measurements in a personalized treatment strategy. These are part of the protective (non-canonical) branch of the RAAS, which reverses the vasoconstrictive and pro-fibrotic actions of angiotensin II. Recent proteomics research has enabled the quantitative measurement of multiple RAAS peptides, including Ang-(1-7), ACE2, and Ang I/II, using liquid chromatography–mass spectrometry. This methodological advance allows for a more accurate assessment of the RAAS balance in children with hypertension and may support early therapeutic decision-making [220]. Longitudinal data also substantiate the clinical effectiveness of RAAS-directed therapy. A 10-year follow-up of children receiving lisinopril, an ACE inhibitor, showed that early treatment reduced systolic blood pressure and proteinuria, particularly in children with renal disease. However, efficacy was lost between 2.5 and 3 years, highlighting the necessity of biomarker-guided monitoring and adjustment of doses over time [221]. Although RAAS inhibitors are widely used for renal protection, their use in pediatric patients, particularly early in disease progression, requires careful consideration of the risks and benefits. The utilization of RAAS inhibitors in pediatrics, particularly where they are initiated early in the disease process, must be carefully assessed for the risk of hyperkalemia, hypotension, and lowered GFR in infants, patients with solitary kidneys, and those with renovascular hypertension [222].

Genetic and phenotypic studies form the basis of this strategy. Polymorphisms of the *ACE* gene, particularly insertion/deletion types, can affect susceptibility to hypertension in children, although their direct impact on RAAS activity is still being investigated [223]. In addition, the enhanced activity of ACE2 in prehypertensive individuals may be indicative of a compensatory mechanism to counteract angiotensin II-induced injury. This strengthens the role of the ACE2/Ang-(1-7) axis as a biomarker and an upcoming therapeutic target [224].

## 6. Conclusions

**Figure 5:**
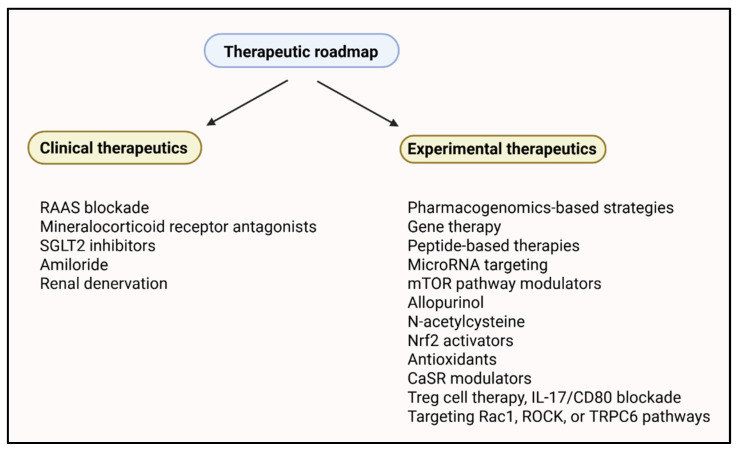
Therapeutic roadmap for hypertensive kidney disease: This figure depicts therapies, both currently available and experimental, organized by their level of clinical development and therapeutic mechanism. The left column identifies therapies established for use in the clinical setting [i.e., renin–angiotensin–aldosterone system (RAAS) inhibition, mineralocorticoid receptor inhibitors, sodium–glucose cotransporter 2 (SGLT2) inhibitors, amiloride (a blocker of the epithelial sodium channel (ENaC)], as well as renal denervation, that are in current use for patients to lower blood pressure and mitigate effects of kidney injury. The right column identifies experimental therapies that are being investigated in preclinical and early-phase studies, including pharmacogenomics and gene or peptide therapies, microRNA antagonists, small-molecule mechanistic target of rapamycin (mTOR) modifiers, and next-generation immunomodulatory interventions (e.g., Treg therapy, interleukin (IL)-17/CD80 blockade). Other agents (e.g., antioxidants, including nuclear factor erythroid 2-related factor 2 (Nrf2) enhancers, mitoquinone (MitoQ), and tempol), calcium-sensing receptor (CaSR) blockers, or any translational modifier of the Ras-related C3 botulinum toxin substrate 1 (Rac1), Rho-associated coiled-coil containing protein kinase (ROCK), and transient receptor potential cation channel, subfamily C, member 6 (TRPC6) axes, are also being studied for any potential effect on slowing disease progression through controlling anti-inflammatory, anti-fibrotic, or oxidative stress pathways.

Hypertensive kidney disease results from a complex interplay of hemodynamic and non-hemodynamic stressors, including aberrant RAAS activity, oxidative damage, immune cell activation, metabolic dysregulation, and epigenetic reprogramming. These mechanisms disrupt glomerular and tubular homeostasis, resulting in fibrosis, proteinuria, and progressive renal impairment [17,18]. Evolutionary precision medicine approaches are beginning to recast the diagnosis of hypertensive renal injury. By applying genetic screening, biomarker qualification, multi-omics profiling, and other methods, clinicians can differentiate between risk-prone individuals and personalize interventions to their individual molecular lesions [21,173]. Therapeutic paradigms are also being redefined (Figure 5). Device-based therapies, such as RDN, have demonstrated not only blood pressure-lowering effects but also reductions in systemic inflammation and oxidative stress, with renoprotection beyond hemodynamic control [174]. In parallel, pharmacological combinations of RAAS blockers, SGLT2 inhibitors, and anti-fibrotic agents have shown additive benefits in slowing disease progression [176,212].

Personalized therapeutic interventions are particularly critical in the pediatric population, in which RAAS activation and immune signal modification early in life can set the course for lifelong kidney vulnerability. The PHRASE trial suggests that molecular phenotyping in childhood can facilitate early, specific interventions that delay or prevent the development of CKD [221]. Together, the integration of molecular diagnostics, new therapeutics, and systems-level analysis represents a paradigm shift towards precision nephrology from generic treatment algorithms. Not only will this enhance the potential to prevent irreversible renal injury, it will also have the potential to increase survival and quality of life along the spectrum of hypertensive kidney disease [214]. As translational research continues to elucidate the multifaceted nature of hypertensive nephropathy, collaborative innovation spanning basic science, clinical observation, and technology will be crucial. While preclinical studies may demonstrate efficacy, several issues must be addressed before they can be integrated into clinical care. First, many of the experimental agents evaluated in preclinical studies, including NLRP3 inhibitors, peptide-based interventions, and gene editing technologies, have unresolved safety concerns related to their long-term use in at-risk patient populations (e.g., pediatric patients and patients with concomitant comorbidities). Second, patient-specific variability in response to targeted therapy is poorly understood, complicating the development of precision medicine protocols. In addition, regulatory impediments (e.g., language of labeling) and investment from manufacturers in developing agents, as well as developing firm biomarkers to securely predict response, may delay pathways to clinical adoption. Ultimately, this course toward individualized, mechanism-based therapy is sure to transform clinical practice, expedite early detection, and optimize patient outcomes.

## Figures and Tables

**Table 1:** Key molecular pathways implicated in hypertensive kidney damage.

Pathway	Mechanism	Key Mediators	Ref
Renin–angiotensin system	Vasoconstriction, fibrosis, inflammation	Ang II, AT1R, aldosterone	[17]
Oxidative stress	ROS-mediated endothelial and tubular damage	NOX enzymes, Nrf2, SOD	[18]
Immune activation	T-cell infiltration and cytokine-driven damage	IL-17, TNF-α, NF-κB	[19]
Mechanical stress	Cytoskeletal disruption in podocytes	TRPC6, Piezo1, actin regulators	[20]
Hypoxia	Capillary rarefaction and HIF pathway activation	HIF-1α, VEGF, ADMA	[18]
Metabolic dysregulation	ATP depletion, substrate shift, energy stress	mTOR, AMPK	[18]
Epigenetics	Altered gene expression via miRNA and methylation	miR-144-3p, miR-129, DNA methyltransferases	[21]

Abbreviations: Ang II, angiotensin II; AT1R, angiotensin II type 1 receptor; ROS, reactive oxygen species; NOX, NADPH oxidase; Nrf2, nuclear factor erythroid 2-related factor 2; SOD, superoxide dismutase; IL-17, interleukin-17; TNF-α, tumor necrosis factor-alpha; NF-κB, nuclear factor kappa-light-chain-enhancer of activated B cells; TRPC6, transient receptor potential canonical 6; HIF-1α, hypoxia-inducible factor 1-alpha; VEGF, vascular endothelial growth factor; ADMA, asymmetric dimethylarginine; mTOR, mechanistic target of rapamycin; AMPK, AMP-activated protein kinase; miR-144-3p, microRNA-144-3p; miR-129, microRNA-129.

**Table 2:** Precision medicine strategies in hypertensive kidney disease.

Strategy	Target	Example Therapy or Tool	Ref
Genetically guided therapy	Gene variants influencing drug response	LSD1 polymorphism → MR antagonist use	[173]
Device-based therapy	Sympathetic nerve activity	Renal denervation	[174]
Biomarker-guided treatment	Inflammation, fibrosis, oxidative stress	IL-6, TGF-β, NGAL, KIM-1	[175]
Combination therapy	Multi-pathway blockade	RAS + SGLT2 + finerenone	[176]
RNA-targeted therapy	Disease-driving miRNA/mRNA	siRNA, antisense oligos	[20]
Systems biology	Patient stratification by omics	Multi-omics platforms, Nephroseq, scRNA-Seq	[21]

Abbreviations: IL-6, interleukin-6; KIM-1, Kidney Injury Molecule-1; LSD1, Lysine-Specific Demethylase 1; MR, Mineralocorticoid Receptor; NGAL, Neutrophil Gelatinase-Associated Lipocalin; RAS, Renin-Angiotensin System; SGLT2, Sodium-Glucose Cotransporter 2; siRNA, Small Interfering RNA; scRNA-Seq, Single-Cell RNA Sequencing; TGF-β, transforming growth factor-beta

## Abbreviations

The following abbreviations are used in this manuscript:

Abbreviation	Full Name
ACE2	Angiotensin-Converting Enzyme 2
ADD3	Adducin 3
ADMA	Asymmetric Dimethylarginine
Ang II	Angiotensin II
AMPK	AMP-Activated Protein Kinase
AP1	Activator Protein 1
ARB	Angiotensin Receptor Blocker
AREs	Antioxidant-Responsive Elements
AT1R	Angiotensin II Type 1 Receptor
Cas9	CRISPR-associated protein 9
CASP1	Caspase 1
CKD	Chronic Kidney Disease
CPT1	Carnitine Palmitoyltransferase-1
CRISPR	Clustered Regularly Interspaced Short Palindromic Repeats
CTGF	Connective Tissue Growth Factor
DPP-4	Dipeptidyl Peptidase-4
ECM	Extracellular Matrix
EMT	Epithelial–Mesenchymal Transition
eGFR	Estimated Glomerular Filtration Rate
ENaC	Epithelial Sodium Channel
eNOS	Endothelial Nitric Oxide Synthase
ER	Endoplasmic Reticulum
ESKD	End-Stage Kidney Disease
FAO	Fatty Acid Oxidation
FGF-23	Fibroblast Growth Factor 23
FGFR1	Fibroblast Growth Factor Receptor 1
FXR1	Fragile X Mental Retardation Syndrome-Related Protein 1
GLP-1	Glucagon-Like Peptide 1
GWAS	Genome-Wide Association Study
HIF	Hypoxia-Inducible Factor
Hsp27	Heat Shock Protein 27
ICAM	Intercellular Adhesion Molecule 1
IL-1β	Interleukin-1 Beta
IL-6	Interleukin-6
IL-10	Interleukin-10
IL-17	Interleukin-17
JAK-STAT	Janus Kinase–Signal Transducer and Activator of Transcription
JNK	c-Jun N-Terminal Kinase
KIM-1	Kidney Injury Molecule-1
L-FABP	Liver-Type Fatty Acid-Binding Protein
MAPK	Mitogen-Activated Protein Kinase
MCP-1	Monocyte Chemoattractant Protein-1
miRNA	MicroRNA
MitoQ	Mitoquinone
MR	Mineralocorticoid Receptor
MRTF-A	Myocardin-Related Transcription Factor-A
mtDNA	Mitochondrial DNA
mTOR	Mammalian Target of Rapamycin
NAC	N-Acetylcysteine
NADPH	Nicotinamide Adenine Dinucleotide Phosphate
NFATc1	Nuclear Factor of Activated T Cells, Cytoplasmic 1
NGAL	Neutrophil Gelatinase-Associated Lipocalin
NF-κB	Nuclear Factor Kappa-Light-Chain-Enhancer of Activated B Cells
NLRP3	NOD-, LRR-, and Pyrin Domain-Containing Protein 3
NO	Nitric Oxide
Nrf2	Nuclear Factor Erythroid 2-Related Factor 2
PAI-1	Plasminogen Activator Inhibitor 1
PHRASE	Pediatric Hypertension and the Renin–Angiotensin SystEm Study
PLC-IP_3_	Phospholipase C-Inositol 1,4,5-Trisphosphate Pathway
PPARα	Peroxisome Proliferator-Activated Receptor Alpha
RAAS	Renin–Angiotensin–Aldosterone System
Rac1	Ras-related C3 botulinum toxin substrate 1
RDN	Renal Denervation
ROCK	Rho-Associated Coiled-Coil Containing Protein Kinase
ROS	Reactive Oxygen Species
RAS	Renin–Angiotensin System
SRF	Serum Response Factor
SGK1	Serum- and Glucocorticoid-Regulated Kinase 1
SGLT2	Sodium–Glucose Cotransporter 2
SHR	Spontaneously Hypertensive Rat
siRNA	Small Interfering RNA
SNS	Sympathetic Nervous System
STAT3	Signal Transducer and Activator of Transcription 3
STING	Stimulator of Interferon Genes
TAZ	Transcriptional Co-Activator
TGF-β	Transforming Growth Factor Beta
TNF-α	Tumor Necrosis Factor-Alpha
Th17	T Helper 17 Cells
TIMP-1	Tissue Inhibitor of Metalloproteinases-1
TonEBP/NFAT5	Tonicity-Responsive Enhancer Binding Protein/Nuclear Factor of Activated T Cells 5
Treg	Regulatory T Cell
TRP	Transient Receptor Potential
TRPC6	Transient Receptor Potential Cation Channel, Subfamily C, Member 6
UMOD	Uromodulin
VCAM-1	Vascular Cell Adhesion Molecule 1
VEGF	Vascular Endothelial Growth Factor
VSMC	Vascular Smooth Muscle Cell
YAP	Yes-Associated Protein
